# When Geometry Constrains Vision: Systematic Misperceptions within Geometrical Configurations

**DOI:** 10.1371/journal.pone.0151488

**Published:** 2016-03-17

**Authors:** Natale Stucchi, Lisa Scocchia, Alessandro Carlini

**Affiliations:** 1 Department of Psychology, University of Milano-Bicocca, Piazza dell’Ateneo Nuovo 1, 20126 Milano, Italy; 2 NeuroMi—Milan Center for Neuroscience, Milan, Italy; 3 Laboratory for Research on Learning and Development (LEAD), CNRS UMR 5022, University of Burgundy, Dijon, France; Centre de Neuroscience Cognitive, FRANCE

## Abstract

How accurate are we in reproducing a point within a simple shape? This is the empirical question we addressed in this work. Participants were presented with a tiny disk embedded in an empty circle (Experiment 1 and 3) or in a square (Experiment 2). Shortly afterwards the disk vanished and they had to reproduce the previously seen disk position within the empty shape by means of the mouse cursor, as accurately as possible. Several loci inside each shape were tested. We found that the space delimited by a circle and by a square is not homogeneous and the observed distortion appears to be consistent across observers and specific for the two tested shapes. However, a common pattern can be identified when reproducing geometrical loci enclosed in a shape: errors are shifted toward the periphery in the region around the center and toward the center in the region nearby the edges. The error absolute value declines progressively as we approach an equilibrium contour line between the center and the outline of the shape where the error is null. These results suggest that enclosing an empty space within a shape imposes an organization to it and warps its metrics: not only the perceived loci inside a shape are not the same as the geometrical loci, but they are misperceived in a systematic way that is functional to the correct identification of the center of the shape. Eye movements recordings (Experiment 3) are consistent with this interpretation of the data.

## Introduction

The catchy slogan "The whole is more than the sum of its parts" has been associated to the Gestalt psychology since its very beginning. Of course, the attempt of summarizing a theory in a slogan entails some over-simplification, and probably the first Gestalt psychologists would have had some complaints about it because their general claim was more elaborated. By using Wertheimer's words we can re-formulate the claim as "The properties of any of the parts are determined by the intrinsic structural laws of the whole" [1,2]. If we restrict our perspective to visual perception, we can say that the perceptual value of any point of a visual shape is a function of its total structure.

This claim was pursued in two very ambitious research programs [3,4]. The first one is mainly associated to Wertheimer and his students, who started to investigate how our visual experience is organized and formulated some principles governing its organization [5,6]. Instances of these principles are similarity, proximity, common fate, closure, symmetry, which can all be traced back to a more encompassing general principle that Wertheimer formulated as the tendency of our perceptual experience to take the simplest structure allowed by the given conditions (Prägnanz). Later Kanizsa rephrased the basic aim of this program as the undertaking to define a grammar of perception [7]: the main results of such a research program are common shared psychological knowledge.

The second research program was mainly sponsored by Köhler [8,9,10]. Köhler's basic idea was that perceived shapes (Gestalten) are like physical systems which, left to themselves, show the tendency to achieve an equilibrium (the sum of the forces of the system is null) or at least a maximal level of stability (the expenditure of energy is minimal). Thus, perceptual Gestalten result from field forces that lead to the simplest organization (Prägnanz). Köhler hypothesized that the neural processes which could account for perceived Gestalten are physical Gestalten in the brain (brain electromagnetic fields) which are isomorphic to the perceived Gestalten (psychophysical isomorphism). The hypothesis of isomorphic neural processes underlying psychological Gestalt phenomena was empirically ruled out in the last mid-century [11,12]. Unfortunately, together with the idea of isomorphic brain electromagnetic fields, the whole vector field approach to visual perception has been discarded. Since then, it has had only an historical value and very rare empirical efforts have been produced in that direction (e.g. [13,14,15]). Instead, we do believe that the vector field framework is still useful to understand perceptual phenomena and the empirical evidence we will present in this work will be discussed within this framework.

Indeed, evidence supporting the vector field framework has been collected in the past [14,15,16,17,18,19,20]. Let us examine the articles of Brown and Voth [16] and of Orbison [17]as instances of how the notion of vector field can be used to investigate visual perception and to explain visual phenomena. Brown and Voth [16] proposed that a perceptual vector field is determined by the action of restraining forces, which account for the stability of directly visible elements of a scene (for instance, the geometrical properties of a shape such as its contour, position, lines, boundaries, angles, and so on), and cohesive forces, which account for the organizing properties of the scene (for instance, symmetry, contiguity, common fate, temporal succession of events, and so on). Any manipulation of cohesive forces should have an effect on the restraining forces and modulate the geometrical properties of an object. The authors showed that the perceived trajectory of an apparent motion can be modified by changing the timing of events in a way that can be predicted by their vector field model. Orbison [17] applied the very same idea to stable configurations: he employed geometrical patterns as organized backgrounds on which he displayed simple geometrical items (shapes and lines). He showed that the patterned background acted as a vector field and induced predictable and systematic perceptual distortions of the items presented within it. More recently, Stadler and colleagues [14] asked participants to reproduce, by successive comparisons, the positions of several points presented one by one on a sheet of paper. The authors observed systematic dislocations toward four attractor areas located next to the four corners. They provided a mathematical description of their results in terms of vectors fields and claimed that the errors they observed are a consequence of force fields acting in visual perception.

If we accept the proposal of Brown and Voth [16] and apply it to a simple shape (e.g: on a sheet of paper, an empty white area delimited by a black line as a circle, a triangle, a rectangle or even an irregular shape) we can associate the restraining forces with the visible geometrical outline of the figure and the cohesive forces with its center, which is not marked by a visible graphic element but it is certainly perceived. These forces make up the vector field of the figure. The outline and the center of the figure interact to maintain the stability of the perceived shape: a systematic distortion of the space between them would be an evidence for the existence of a perceptual force field, intended as a vector field that describes non-contact forces acting at various loci in space. The central aspect of a perceptual Gestalt interpreted as a vector field is that every loci of the Gestalt interact with all the other loci.

A previous study aimed to measure the perceptual distortion of the one-dimensional space defined by a horizontal line [21]. It assessed perception of 15 target loci pinpointed on the line by another small vertical line intersecting it, by means of a position reproduction task. Given that previous experiments on points localization insisted on the role of memory in generating space errors [14,20], the same task was performed both in a successive comparison experiment (target and adjustable stimuli were presented one after the other at the same position in space) and in a simultaneous comparison experiment (both the target and the adjustable stimulus were presented at the same time but at different positions in space). The results showed a systematic modulation of the error made in estimating the target positions. Null reproduction errors were found for the center of the line and for two symmetrical positions between the center and the edges. Furthermore, null errors were presumed for the reproduction of positions corresponding to the edges, which would work as visible landmarks. For all the other positions errors were not null, rather, positions were systematically reproduced away from the center and the endpoints. The same reproduction error pattern was present both in the successive and in the simultaneous comparison experiments. The only clear difference between the two procedures was a systematic decrease of the error size with the successive comparison experiment. Overall, the systematic modulation of the localization error was interpreted as the outcome of the process of identifying the perceptual center of the line.

The results obtained for the line, together with the above mentioned consideration that the structure imposed to the space by a shape may entail the action of perceptual force fields, drove us to start a new study aimed to assess possible perceptual distortions of the two-dimensional space defined by a simple shape. In the present study we employed geometrical shapes outlines presented on a uniform background (a circle and a square outline) and asked participants to reproduce the position of a previously seen point within the shape by means of a mouse cursor. We computed the difference between the geometrical loci and the corresponding perceived loci as a measure of misperception. We predicted that the space delimited by the shape is not perceptually homogeneous but warped as the one-dimensional space defined by a line [21]. In the case of simple shapes outlines, the salient perceptual elements that are expected to determine the warping of the two-dimensional space, possibly acting as cohesive and restraining forces of a vector field, are the center and the contours. Obviously, no error is expected for the points lying on the outline, which would serve as a visible landmark in the reproduction task. By analogy with the previous experiment on the line [21], minimal error is also expected when reproducing the center. Furthermore, if the center and the edge of the circle exert a repulsive effect on the nearby points[21], the points near the center should be displaced toward the periphery while the points near the edge should be displaced toward the center. At some place between the center and the edge the opposite repulsion effects must achieve an equilibrium point with an error equal to zero.

In other words, we predict to observe a systematic pattern of errors in reproducing the internal loci of a figure: this would be evidence of a perceptual distortion of the area inside a simple shape, determined by the organization the shape imposes to the space it encloses.

## Experiment 1: Circle

### Methods

#### Participants

Six right-handed participants (5 women) with a mean age of 21± 0.7 participated in Experiment 1.

Participants were undergraduate students at the University of Milano-Bicocca and took part in the experiment in exchange for course credits after having provided their informed written consent. All participants were naive to the purpose of the study and reported normal or corrected-to-normal visual acuity. The tenets of the Declaration of Helsinki were observed and the study was approved by the Ethics Committee of the University of Milano-Bicocca.

#### Stimuli and Apparatus

Stimuli were presented on a 20-inch Philips Brilliance 202P7 Monitor (with a vertical refresh rate of 75 Hz and resolution of 1600 x1200 pixels, where a pixel is a 0.28 mm side square), interfaced with an Asus Intel® Core^TM^2 Quad 2.40 GHz personal computer equipped with a NVIDIA® GeForce® 9500 GT Video Board. Stimuli were handled and displayed using Matlab and the Psychophysics Toolbox 3 extensions for Matlab [22,23,24]. A black 680x1000 mm reduction screen with a circular hole (280 mm in diameter) cut out at its center was fixed in front of the computer monitor to eliminate any possible view of the surrounding monitor frame and to minimize possible edge interferences.

**Fig 1:**
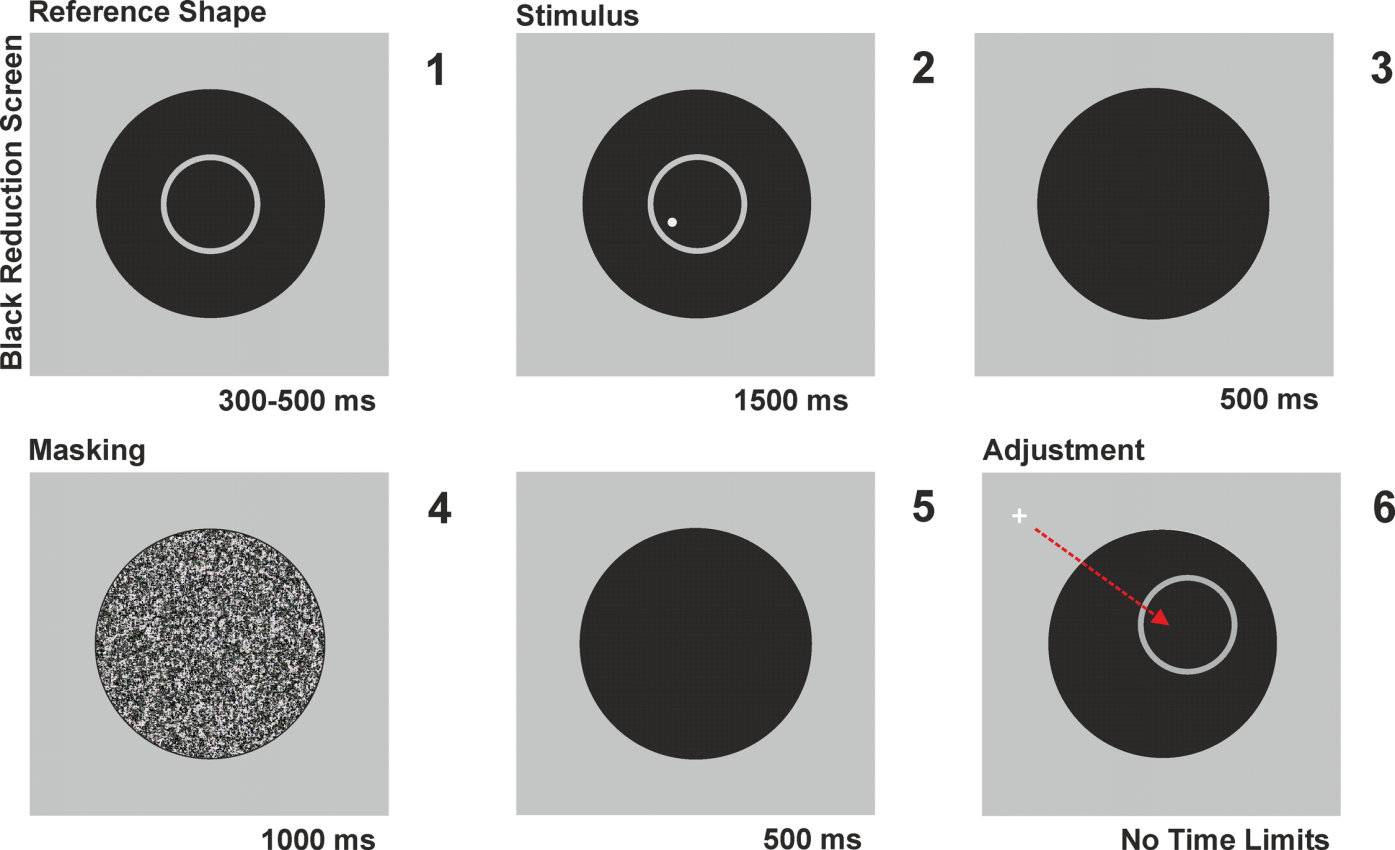
Trial sequence. Observers viewed the stimuli through a black 680x1000 mm reduction screen with a circular hole (280 mm in diameter) cut out at its center and fixed at 6 cm in front of the computer monitor. Each trial began with a homogenous black screen lasting for 1200–1800 ms (meaning that the actual duration was randomly chosen in the interval). Then the circle outline (diameter: 16.3 degrees of visual angle) was presented (panel 1) and 300–500 ms later the small target disk was added to the display (panel 2). The whole stimulus (circle plus target) remained visible for 1500 ms. It was followed by a black screen for 500 ms (panel 3), and, then, by a masking screen for 1000 ms (panel 4). The screen went black again for 500 ms (panel 5), and then only the circle was displayed with a position jitter (with coordinates randomly chosen in an interval of ±3 degrees of visual angle independently on the X and Y axes). Afterwards, a crosshair cursor was displayed behind the reduction screen in a screen quadrant that did not contain the target. Participants were required to move the cursor inside the visible area in order to reproduce the previously seen target position: they clicked the mouse once they had achieved the exact match (panel 6). Stimuli are not drawn to scale.

Stimuli were presented on a black background (0.16 cd/m^2^): they consisted of a light gray circle outline (35 cd/m^2^) and of a small white disk (diameter 1.7 mm or 0.2 degrees of visual angle, 108 cd/m^2^) presented as a target inside the circular shape. The circle outline was 140 mm in diameter, which corresponds to about 16.3 degrees of visual angle at 43 cm viewing distance. Its edge was drawn by a 1 pixel thick line, and smoothed by the Psychophysics Toolbox anti-aliasing system. The small target disk was randomly presented at one of 57 possible points inside the circle as planned out in the experimental design (Fig 1).

#### Procedure and Design

Participants sat in a dark quiet room at 43 cm in front of the computer screen, with their head movements constrained by a chin and forehead rest. They were informed that each trial was composed by two steps: in the first step they would be presented with a target (a small disk) enclosed in a circle; and then, in the second step, when the target had vanished, they would have to place a cross-hair cursor at the exact position where they had previously seen the target by means of the computer mouse.

The trial sequence is illustrated in Fig 1. Each trial began with a homogenous black screen lasting for 1.2–1.8 seconds (meaning that the actual duration was randomly chosen in the interval). Then the circle outline was displayed at the center of the screen and 0.3–0.5 s later the disk target was added to the display. The whole stimulus (circle plus target) remained visible for 1.5 s. It was followed by a black screen for 0.5 sec, and, then, by a masking screen for 1 s. The full screen masking image was composed by a white-noise luminance square distribution, and was meant to erase any visual after-image. The screen went black again for 0.5 s, and then only the circle was displayed at a position that varied from the previously seen circle between -3.3 and + 3.3 degrees of visual angle (randomly and independently on the X and Y axes). This position jitter aimed to avoid that participants employed any sort of fixed external reference rather than the actual displayed circle: participants were explicitly told that the second circle could be displayed at a different position compared to the first, and that they had to reproduce the disk position relative to the circle. Afterwards, a crosshair cursor appeared in a screen quadrant that did not contain the target as soon as participants moved the computer mouse: the participants’ task was to reproduce the previously seen target position *within the circle* by moving the cursor and clicking the mouse once they had achieved the exact match. Afterwards, a new trial started. Participants were allowed to take a break every 10 trials.

**Fig 2:**
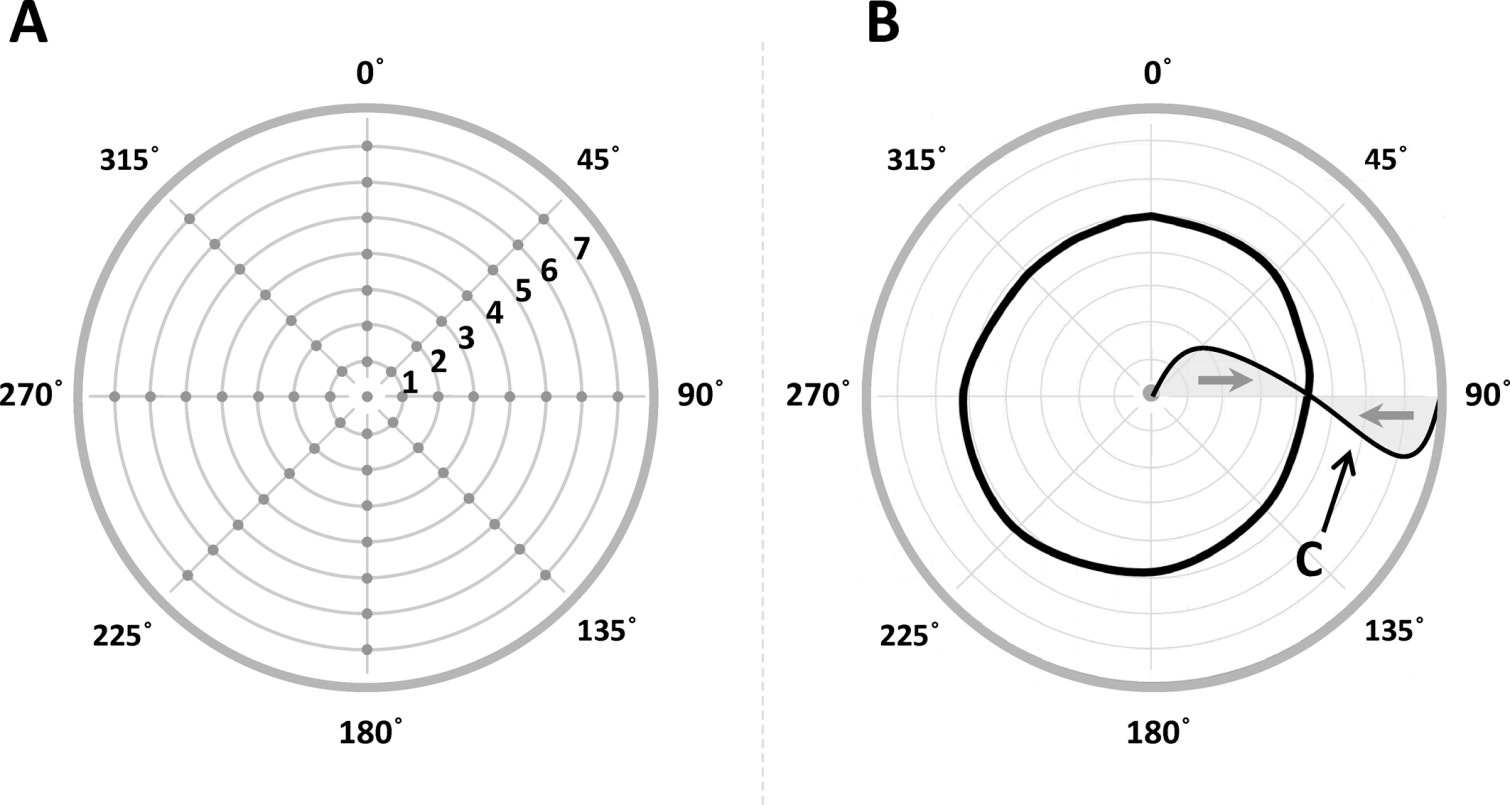
Experiment 1: Experimental design, expected and observed results. *Panel A* illustrates the design used in the experiment. 57 points belonging to the inner area of a circle were presented. 56 points were distributed along 8 radii (from 0° to 315° by steps of 45°) at 7 equally-spaced locations for each radius (labels 1 to 7). The remaining last point coincided with the center of the circle. Inner lines and points are reported here for illustrative reasons and were not displayed during the experiment, where only the empty circular outline was presented. *Panel B*. 57 x 9 estimations were produced in a random order (9 replicated estimations per point). Obviously, no radial error is expected for the points located on the circumference. Therefore, points lying on the circumference were not included in the experimental design. Minimal error was also observed at the center. If the center and the edge of the circle exert a repulsive effect on the nearby points, the points near the center should be displaced toward the periphery (positive error) while the points near the edge should be displaced toward the center (negative error). At some place between the center and the circumference the opposite repulsion effects must achieve an equilibrium point with an error equal to zero. These expected results are illustrated by the curve **C** which represents the hypothetical modulation of the estimation error between the center and the periphery. The black curve represents the empirical contour line of null error actually estimated by participants in Experiment 1.

The experimental design is illustrated in Fig 2A. 57 points inside the circle were tested in the experiment. 56 points were distributed along the 8 radii (from 0 degrees to 315 by steps of 45 degrees) in 7 equally-spaced locations for each radius (labels 1 to 7 in Fig 2A). The remaining last point coincided with the center of the circle. The estimation of each point was replicated 9 times. So, the whole experimental design consisted of 57 points (8 radii x 7 locations plus the center) by 9 repetitions, which gives a total of 513 trials, which were presented in three separated sessions of about 40 min (57 points by 3 repetitions per session, randomly displayed) and held at least two hours apart, within a 10 days interval.

### Data analysis

In line with the experimental hypotheses, our dependent variable of interest was the radial error of each participant’s responses in the position reproduction task. In fact, possible perceptual mislocalizations towards the centre or towards the edges of a figure are captured by the radial error. For completeness however, we report both the radial and the tangential error data in the Results sections. The points on the circumference were not included in the experimental design because the observers were expected to commit no radial errors for them, being the circumference displayed on the screen as the frame of reference relative to which participants provided their responses. Hence, we assumed null radial error at the circumference. Generalized Linear Model (GLM) analyses with Radius (8 levels) and Location (7 levels) as within-subjects factors were performed on radial and tangential estimation errors. The 9 repetitions were collapsed into one single average value. Whenever the GLM analyses highlighted a significant main effect or interaction, Bonferroni post-hoc tests were used to compare experimental conditions—i.e., all the possible pairwise comparisons were tested for all the factor (or interaction) levels. The significant comparisons reported in the text refer to Bonferroni post-hoc tests that meet at least the 5% significance level (p < .05). Statistical analyses were conducted using Matlab (Mathworks, Natick, MA) and Statistica (Statsoft, Italy).

### Results

Fig 2B gives a first overview of results. Circumference, center, and the middle circular line between them must be interpreted as contour lines representing a null radial estimation error (i.e. the displacement along the direction of the radius to which the point belongs). Inside the region comprised between the center and the middle line participants make an overestimation error toward the periphery (positive error by convention), while in the annular region between the middle line and the circumference they make an underestimation error toward the center (negative error). The fact that the center has a special status and that observers can reproduce its position with minimal error has been shown within the framework of a single line [21]. Here as well participants are remarkably good in estimating the center of a circle: they do show a systematic bias, which is however very small and consistent across observers (x-error: -1.5 pixels ±1.2 SE, y-error: 4.3 pixels ±3.2 SE).

**Fig 3:**
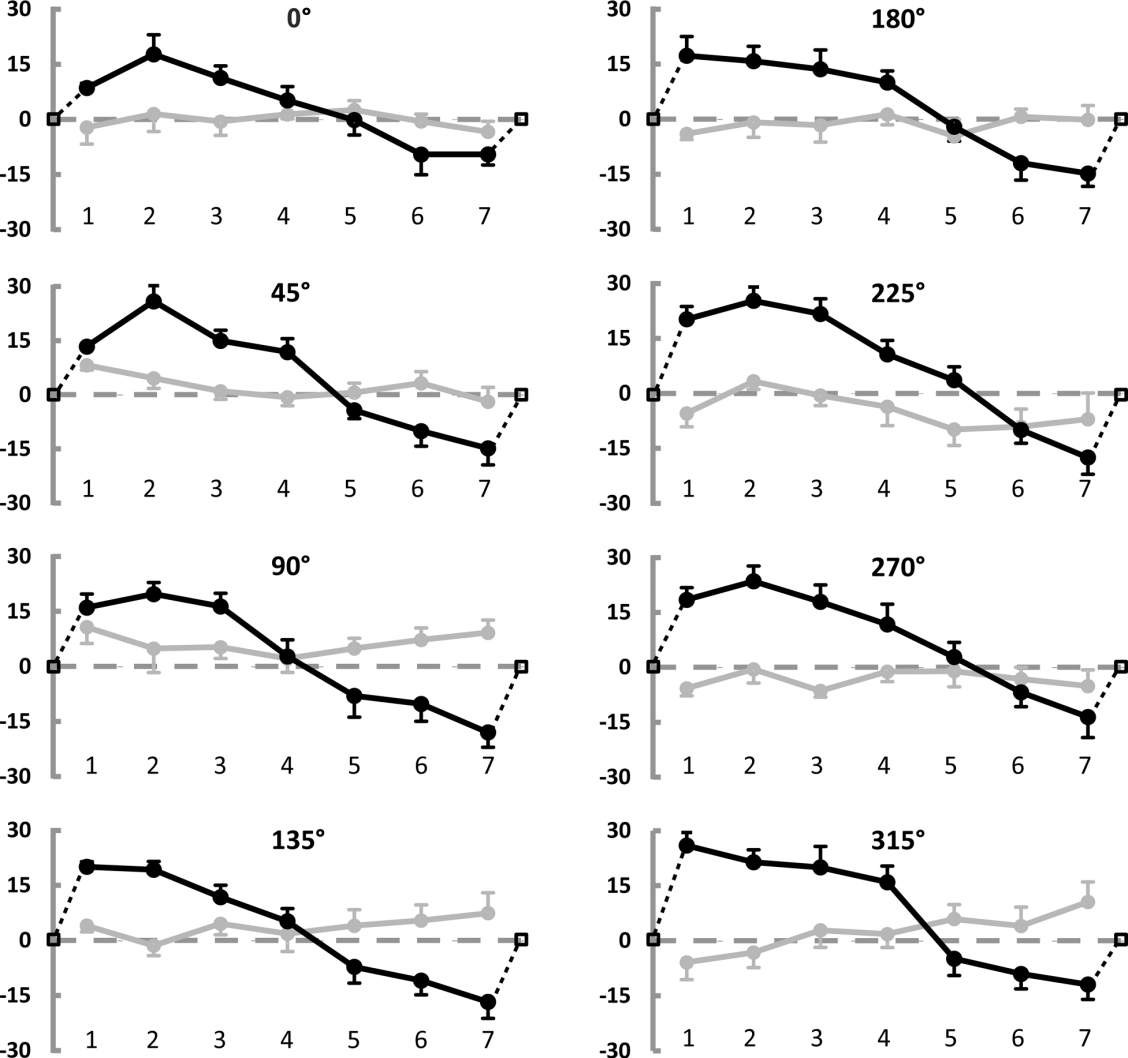
Results of Experiment 1: Modulation of radial and tangential errors. Average Constant Errors (CE) made when reproducing points locations (7 points by 8 radii, see Fig 1A), expressed in pixels. Black curves represent the radial component of the CE (i.e. the displacement along the direction of the corresponding radius). Positive values indicate displacement toward the periphery, negative toward the center. Black dotted lines and empty squares illustrate the expected null radial error at points placed on the circumference and at the center (respectively after 7 and before 1). Grey curves represent the tangential component of the CE (i.e. the lateral deviation from the radius direction, orthogonal to it). Positive values refer to clockwise deviations. Error bars represent the standard error of the mean.

Fig 3 shows the general results in a more detailed way: it illustrates the modulation of the radial error (black line) along the seven positions for each of the 8 radii. Positive and negative errors indicate respectively that participants made a displacement error toward the periphery and toward the center while reproducing the loci within the circumference. The expected null error at the circumference and the error in estimating the center (which has been assumed to coincide with zero in the figure for graphical reasons) are included. The error reproduction pattern is very similar for all the radii and remarkably consistent across participants: from the accurate estimation of the center there is a radial displacement error toward the periphery which reaches a maximum and then gradually decreases up to cross the zero. From here on, the error changes sign and goes in the direction of a displacement toward the center, reaches a minimum and ends again with a zero value at the edge. Fig 3 also illustrates the modulation of the tangential error (i.e. the deviation from the radius direction along to the direction orthogonal to the radius) as a grey line. The tangential error is considerably smaller than the radial error (mean radial error: 5.26 pixels ±5.80 SE; mean tangential error: 0.63 pixels ±1.50 SE) and does not show any systematic trend which can be visually appreciated.

The statistical analyses confirm what can be seen in Fig 3. As far as the radial error is concerned, the factor Location results to be significant (F(6,30) = 57.7, p<0.001, partial η^2^ = .92) and explains a remarkably large amount of variance. 14 out of the 21 possible pairwise comparisons are significant. The remaining 7 non-significant comparisons are mainly about adjacent locations (pairs: 1–2, 2–3, 4–5,4–7, 5–6, 5–7, and 6–7), where the error modulation is smaller (see Fig 3). The interaction of Radius by Location (F(42,210) = 1.88, p<0.01, partial η^2^ = .27) also reaches statistical significance: if we look at the Bonferroni post-hoc tests, we observe that 754 out of 1540 tests (49%) reach statistical significance. The overall 1540 comparisons can be divided into three categories: comparisons between corresponding locations at different radii (196), comparisons between non-corresponding locations at a same radius (147) and comparisons between non-corresponding locations at different radii (1197). Only one of the comparisons between corresponding locations at different radii reached significance (radius 0° vs 315° for location 7). Thus, the interaction effect is driven by comparisons between non corresponding locations: of these, 94 mirror the main effect of Location (different locations on a same radius) and the others refer to non-corresponding locations at different radii. As shown in Fig 3, if any systematic effect could be described on the basis of the significant comparisons across non corresponding locations of different radii, it could be interpreted as due to slight differences (in the range of ±3 pixels) around the main large trend given by the factor Location (which varies between -15 and 21 pixels).

As noted above, the tangential error is overall negligible, however the interaction of Radius by Location(F(42,210) = 1.67, p<0.05, partial η^2^ = .25) reaches significance, consistent with small fluctuations along the levels of the main factors (see Fig 3).

## Experiment 2: Square

### Methods

Six naive right-handed participants (5 women) volunteered for Experiment 2. They had normal or corrected to normal vision and their mean age was 25 ± 1.9 None of them had already participated in Experiment 1.

**Fig 4:**
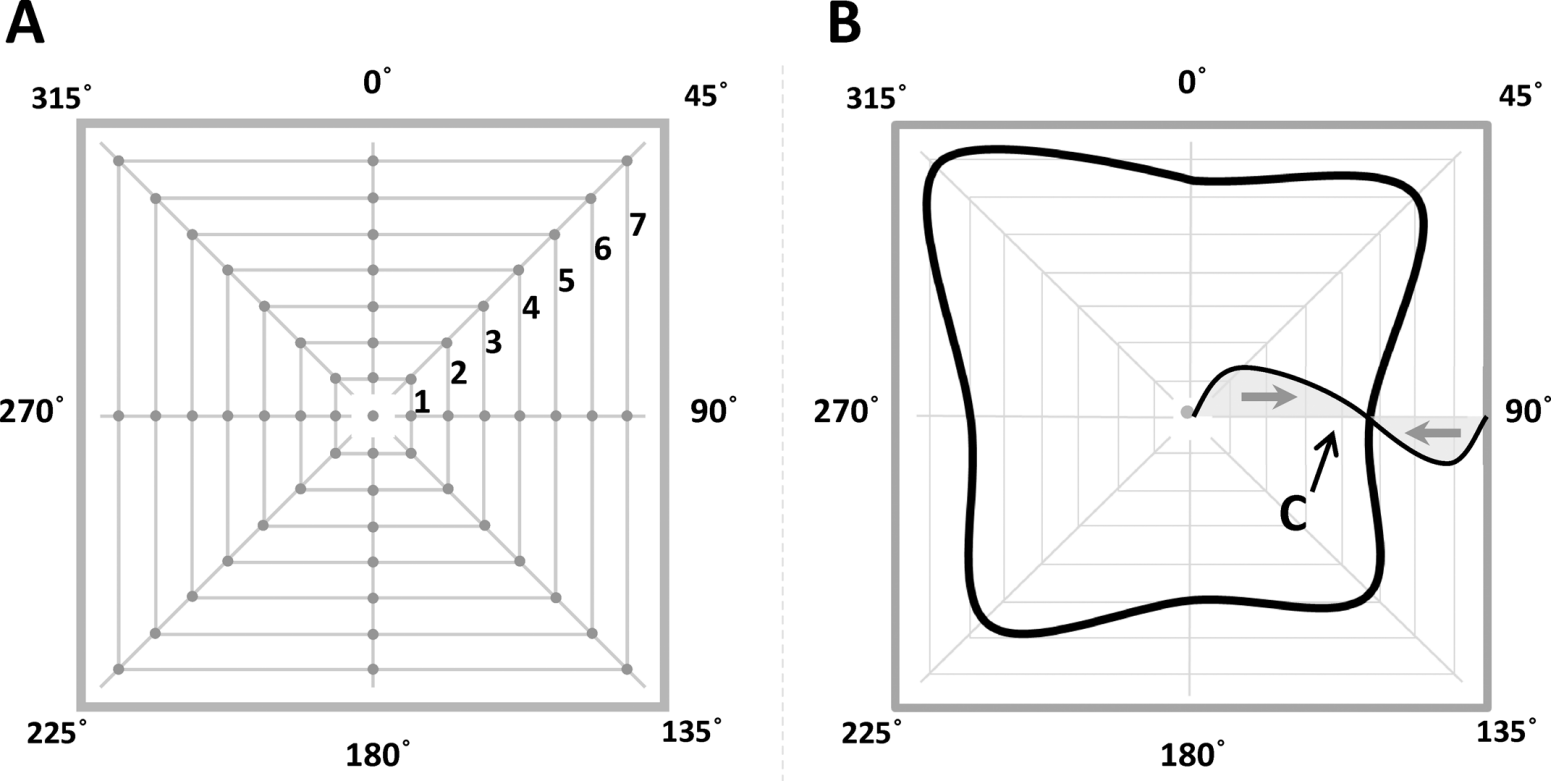
Experiment 2: Experimental design, expected and observed results. *Panel A* illustrates the design used in Experiment 2, which is identical to that used in Experiment 1: 57 points belonging to the inner area of a square (side length: 16.3 degrees of visual angle) were presented. 56 points were distributed along 8 radii (from 0° to 315°) at 7 equally-spaced locations for each radius (labels 1 to 7). The remaining last point coincided with the center of the square. *Panel B*. The experimental procedure was the same as in Experiment 1. As in Fig 1B, expected results are illustrated by the curve **C** which represents the hypothetical modulation of estimation error between the center and the periphery. The black curve represents the empirical contour line of null error actually estimated by participants in Experiment 2.

Apparatus, stimuli and procedure were the same as in the previous experiment. The only substantial difference was the shape used as reference frame, which was a square (side length of 500 pixels, corresponding to 16.3 degrees of visual angle) instead of a circle. Fig 4A illustrates the position of the 57 locations tested in the experiment.

### Results

**Fig 5:**
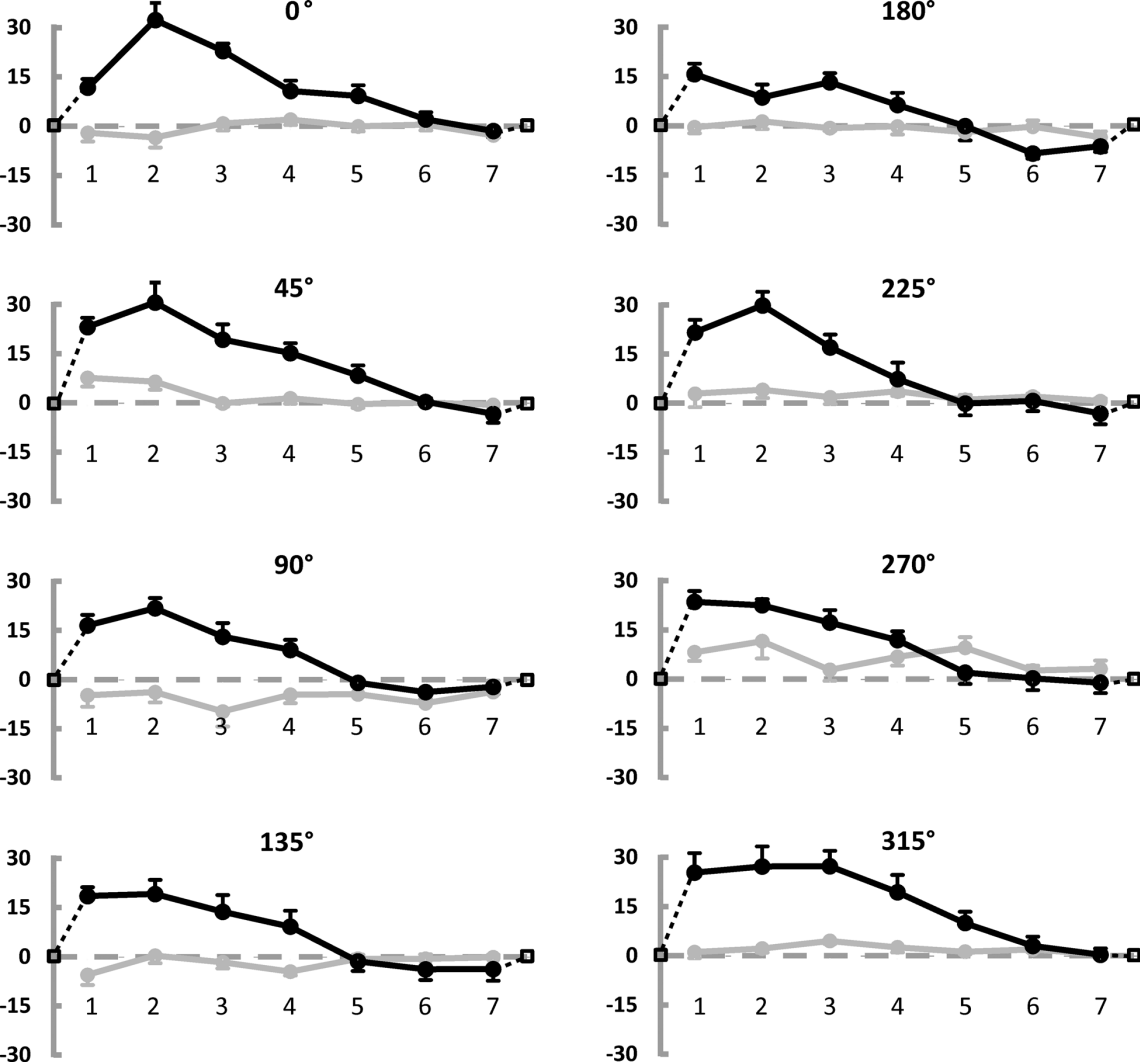
Results of Experiment 2: Modulation of radial and tangential errors. Average Constant Errors (CE) made when reproducing points locations, expressed in pixels. Fig 4 follows the same conventions adopted in Fig 2.

Fig 4B gives a first overview of results. The same conventions adopted in Fig 2B are used here. The contour line representing the null radial error between the edge and the center of the square has a different shape but it is always present. In Fig 5 a remarkably similar trend characterizing the modulation of radial error along the 8 radii and across the six participants can be appreciated. As observed for the circle, the tangential error is very small and the radial error follows a specific pattern: the center is relatively accurately estimated (x-error: -2.65 pixels ±2.42 SE, y-error: 7.47 pixels ±1.68 SE), then the inner loci are reproduced with a positive error (meaning displacement towards the periphery), which reaches a maximum and gradually declines, crosses the zero and changes sign before crossing the zero again in correspondence with the shape edges. If we compare these results to the ones obtained for the circle, we could describe the loci within the shape as perceptually repelled from the center and from the edges. The repulsion from the center is greater than the repulsion from the edges, especially in the case of the square, where the edges appear to have only a limited repulsive effect on the neighbouring loci. As a consequence, the zero contour line inside the square and the circle is modulated in a shape-specific manner. The results of statistical analyses are similar to those we have already described for Experiment 1.

As regards the radial error, the factor Location is significant as expected (F(6,30) = 52.3, p<0.001, partial η^2^ = .91) and explains a very large amount of variance, as observed for the circle. 14 out of the 21 possible pairwise comparisons reached statistical significance, as in Experiment 1. The remaining 7 non-significant comparisons (pairs: 1–2, 1–3, 2–3, 4–5, 5–6, 5–7, and 6–7) are mainly about adjacent positions where error modulation is smaller (see Fig 5).

The main effect of Radius (F(7,35) = 5.1, p<0.001, partial η^2^ = .50) also reaches significance. The Bonferroni post-hoc comparisons show that the effect of Radius is mainly due to the 180° vertical radius where the radial error is systematically smaller than elsewhere, particularly than at 0°, 45° and 315° (Fig 5). Furthermore, the error at 315° is systematically larger than at 180°, 135° and 90°. This asymmetry between the error in the lower right quadrant and the error at 315° might be related to specific reading habits (the square could hint at a page of paper where the upper left angle is particularly salient for Italian readers, as it represents the starting point for reading). However, an explanation of the observed asymmetry in these terms remains speculative at this stage of research.

Finally, the interaction of Radius by Location (F(42,210) = 2.35, p<0.01, partial η^2^ = .32) is significant. As in Experiment 1, the effect is mainly driven by comparisons between non corresponding locations: 654 out of 1540 tests (42.5%) reach statistical significance. Of these, only 9 refer to corresponding locations at different radii: the pair of radii 0°-225° differed at location 7; the pairs of radii 180°-0°, 180°-45°, 180°-225°, 180°-270°, 180°-315° differed at location 6; finally the pairs of radii 315°-90°, 315°-135°, 315°-180° differed at location 5. As shown in Fig 5, the significant comparisons between corresponding locations mainly come from location 6 of radius 180°, and from location 5 of radius 315°, which are different from some other radii, as described above. 77 of the comparisons between non-corresponding locations mirror the main effect of Location (different locations of a same radius). The others refer to non-corresponding locations at different radii: if any systematic effect could be described for this latter type of comparisons, it could be attributed to slight fluctuations (±3 pixels) around the main large trend given by the factor Location (which varies between -3 and 24 pixels, see Fig 5).

In line with Experiment 1, the observed tangential error is very small (less than 2 pixels on average, about 0.5 mm) and the interaction of Radius by Location (F(42,210) = 1.157, p<0.05, partial η^2^ = .24) reaches significance, due to small fluctuations across the large number of interacting levels. Moreover, the main factors Radius (F(7,35) = 5.04, p<0.01, partial η^2^ = .50) and Location (F(6,30) = 3.30, p<0.05, partial η^2^ = .40) show a significant effect. The main effect of Radius is primarily due to the radius at 270°, where the tangential error was systematically different than at 90° and 135°. Instead, the effect of Location can be attributed to small fluctuations in the range of ± 2 pixels (Fig 5).

## Experiment 3: Eye Movements

Experiment 1 and 2 were purposively conducted in free viewing conditions, in order to assess how the circle and the square figures organize the space they delimit in an ecological position reproduction task were no constraints were posed on eye-movements. Under these conditions, the deployment of overt attention by eye movements may substantially vary across different observes. It is well established that spatial resolution is higher when a visual target appears at foveal locations (e.g.[25]) and, moreover, that allocation of attention enhances spatial resolution at its locus [26]. A recent study [27] also showed that allocation of both overt and covert attention attracts the subjectively perceived midpoint towards the attentional focus in a bisection task. It is therefore possible that the voluntary deployment of visual attention affects localization of visual objects. In order to assess the possible contribution of overt attention to the observed warping of the space enclosed by a figure, in Experiment 3 we measured eye-movements during the task.

### Methods

#### Participants

A new pool of six naïve right-handed participants (6 women, mean age 21 ± 2.8) with normal vision took part in the experiment in exchange for course credits after having provided their written informed consent.

#### Apparatus and procedure

Participants sat 66 cm away from a 27-inch LCD monitor (Acer® HN274H; Resolution: 1600x900 pixels; Refresh rate: 60 Hz) with their head movements constrained by a chin and forehead rest. The monitor was interfaced with an AMD Athlon™ Dual Core 2.00 GHz personal computer equipped with a NVIDIA® GeForce® GTX 560 Video Board. The experimental apparatus included an infrared remote/head-free eye-tracking system (EyeLink 1000®, SR Research Ltd.) with a nominal recording spatial resolution of 0.01 degrees of visual angle and a sampling rate of 1000 Hz. A black 680x1000 mm reduction screen with a circular hole (280 mm in diameter) cut out at its center was placed at 6 cm in front of the computer monitor to eliminate any possible view of the surrounding monitor frame and to minimize possible edge interferences.

Stimulus parameters (size and luminance) were adjusted in order to replicate the stimulus conditions of Experiment 1. The experimental procedure was the same as in Experiment 1, with the single exception that only the 15 points on the horizontal axis of the circle (i.e. the points lying on Radius 90° and Radius 270°, see Fig 2A) were presented 9 times each. Overall participants took about 50 minutes to complete Experiment 3, including two compulsory breaks of 5 minutes each, after which the system was recalibrated.

### Results

**Fig 6:**
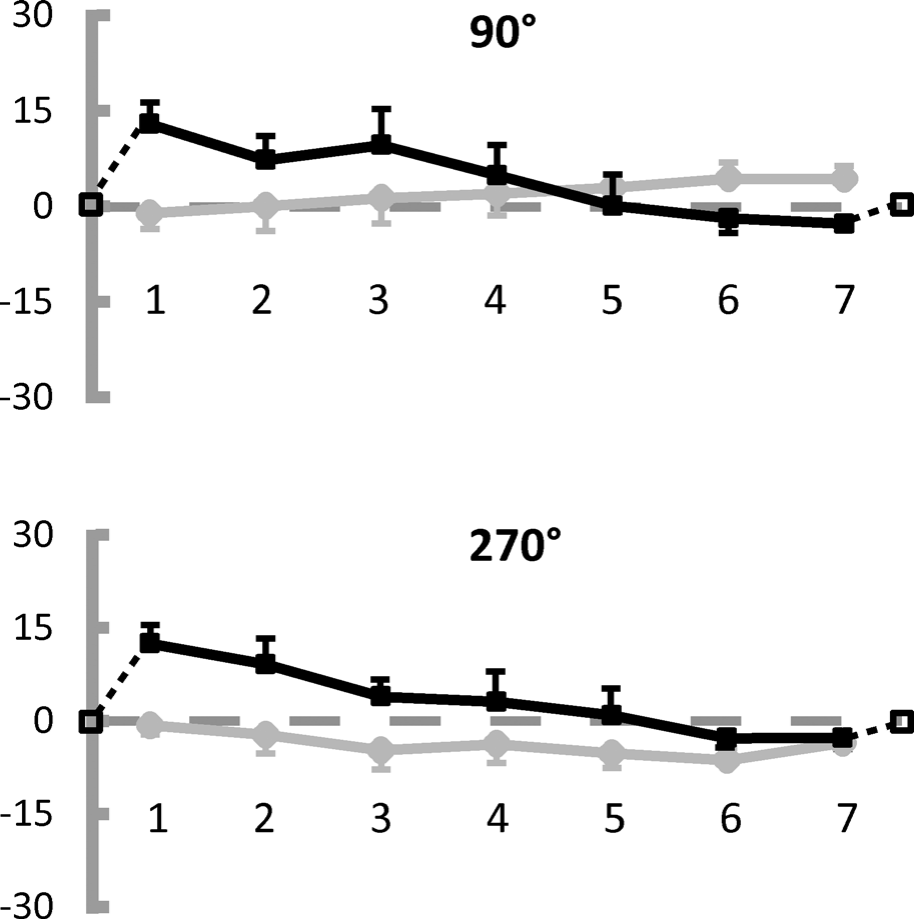
Results of Experiment 3: Modulation of radial and tangential errors along the horizontal diameter. Average Constant Errors (CE) made when reproducing points locations along the horizontal diameter (7 points by 2 radii, see Fig 2A), expressed in pixels. The same conventions used in Fig 3 are adopted here.

Fig 6 illustrates the modulation of the radial error (black line) along the seven locations for each of the 2 horizontal radii of the circle (90° and 270°). The reproduction error pattern is remarkably consistent across participants, very similar for both the radii and analogous to the corresponding radii already tested in Experiment 1, which underlines its reproducibility. Besides, participants are remarkably good at estimating the center of the circle: (the average error amounts to 1.45 pixels ±1.5 SE on the horizontal dimension). Fig 6 also illustrates the modulation of the tangential error as a grey line. As in Experiment 1, the tangential error is much smaller than the radial error (mean radial error: 3.83 pixels ±3.24 SE; mean tangential error: 0.94 pixels ±2.72 SE) and does not show any systematic trend which can be visually appreciated.

Generalized Linear Model (GLM) analyses with Radius (2 levels) and Location (7 levels as within-subjects factors were performed on radial and tangential estimation errors. The statistical analyses confirm what can be seen in Fig 6. As far as the radial error is concerned, the factor Location results to be significant (F(6,30) = 6.12 p<0.001, partial η^2^ = .55) and explains a large amount of variance. 4 out of the 21 possible pairwise comparisons are significant (pairs: 1–5, 1–6, 1–7, 2–7): they all refer to comparisons between locations far apart (see Fig 6). No other factors nor interactions are significant. As in Experiment 1, the tangential error is overall negligible and none of the factors nor of the interactions reaches statistical significance.

**Fig 7:**
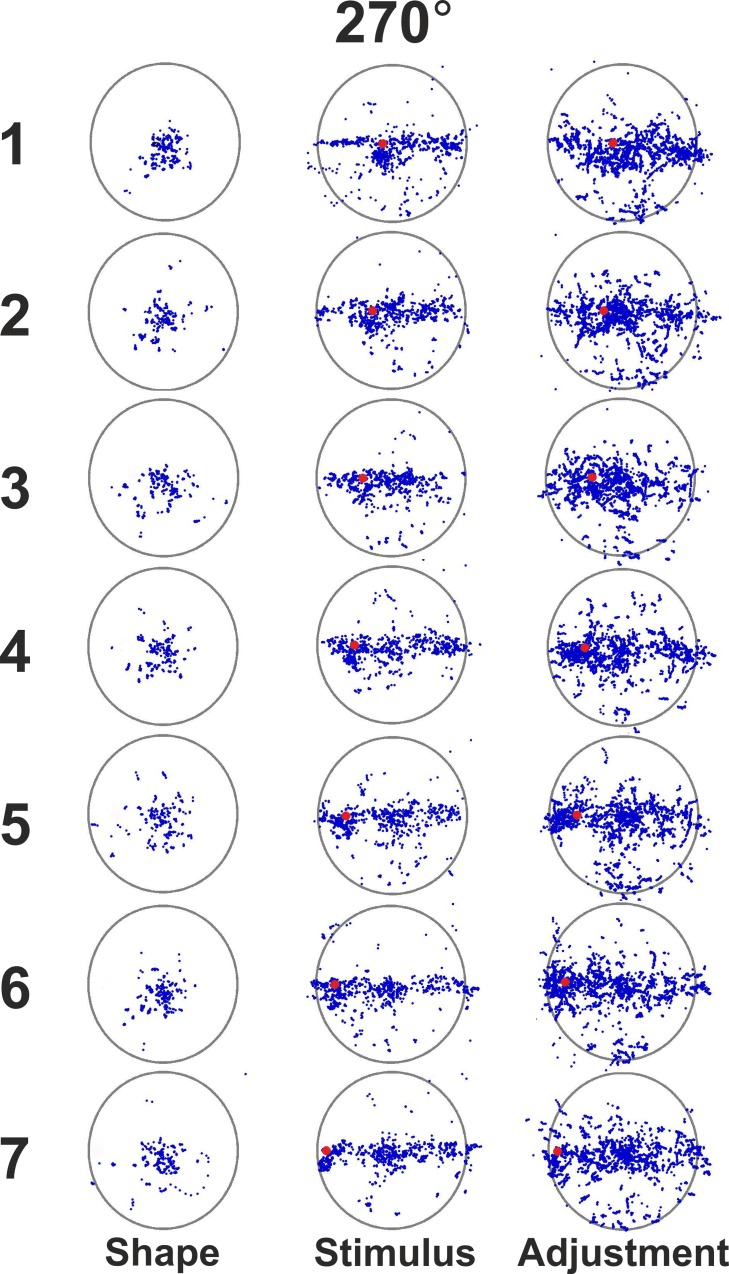
Results of Experiment 3: Eye Movements. Eye movements were originally recorded with a sampling rate of 1000 Hz: only a subset of the recordings (1 every 50 points) is plotted in blue in order to show mainly fixations. Each panel shows recordings of 6 participants by 9 repetitions during the given presentation time (see Fig 1). Only eye movements for the horizontal radius labeled 270° are shown. Each row refers to a different position of the target disk (from 1 to 7). The 3 columns refer to the main phases of each trial sequence as illustrated in Fig 1: Reference Shape presentation (300–500 ms), Stimulus presentation (1500 ms), and Adjustment (no time limits).

Fig 7 illustrates the scattering of eye-movements during the three sensible phases of Experiment 3 (presentation of the shape alone, presentation of the shape with the target, and adjustment/response phase). In order to make the figure readily decipherable only a subset of the sampled eye positions were plotted (1 every 50 points) so that mainly fixations are actually shown. Thus in each panel the recordings of 6 participants by 9 repetitions during the given presentation times (see Fig 1) are visible. Only eye movements for the horizontal radius labeled 270° are shown. The scattering of fixations for the other horizontal radius tested in this experiment are similar to those shown in the figure. At a first visual inspection of the 21 panels, it clearly emerges that participants systematically look inside the inner space delimited by the circle. Besides, during the first phase (Fig 7, Shape panel, first column), when only the circle without a target is presented, participants always look at the center (or nearby the center) of the circle. When the target disk is displayed (red spot in Fig 7 - Stimulus panel, second column), clearly participants not only look at the target, but also inspect the whole horizontal diameter. This is a typical behavior of all the participants, and is consistent with the strategy of representing the position of the target relative to geometrical landmarks as the center and the boundaries of the circle delimiting the horizontal diameter. Indeed, the horizontal scanning strategy was favored by the fact that all stimuli were displayed on the horizontal diameter in this experiment, which emphasized the perceptual saliency of the horizontal boundaries. After the experiment, some participants spontaneously reported to have attempted to assess the target distance from the boundaries and the (non visible) center in order to perform the adjustment task in the final phase of the trial. Finally, fixations in the adjustment phase (Fig 7, Adjustment panel, third column) are more variable than those represented in the second column, mainly because of longer recording times. Still, eye movements in this third phase are not at variance with those of the previous phase, but they show a substantial continuity in the adopted exploratory strategy, which mostly focuses on the whole horizontal diameter.

## Discussion

The results of the three experiments presented here can be summarized as follows: first, the space delimited by a circle and by a square is not homogeneous. Second, the observed distortion appears to be different and specific for the two tested shapes, but it also appears to follow a rule: observers’ responses are shifted toward the periphery in the annular region nearby the center and toward the center in the region nearby the edges. The error absolute value declines progressively as we approach an equilibrium zone where the error is null (Figs 2B and 4B). Third and final, the perceived warping of the space enclosed by a figure cannot be exhaustively explained by allocation of visual attention.

The present results broaden the findings of a previous study [21] where the perceived metric of a line was investigated (see Introduction): observers made systematic errors that could reach the 2.5–7% of the line length, depending on the position along the line to be reproduced. In a control experiment it was established that the error modulation has a perceptual origin and does not depend on memory distortions intervening between the stimulus and the response. Observers reproduced positions nearby the center and nearby the edges systematically away from them, as if the line center and edges exerted a repulsion effect on the region around them: the observers’ error pattern was nicely fitted by the composition of the first two natural harmonics of the line length. The authors proposed that the systematic modulation of the error could be accounted for by a symmetry-detection mechanism which automatically triggers the identification of the midpoint and alters the metric structure of the line. To find the center of a line the observer must use the ends of the line as references: their position can be estimated very accurately given that they are visible elements of the image. The authors suggested that accurate estimation of center and ends of the line is the result of a mechanism that stresses the differences between these salient points and their nearby surroundings. In other words, as soon as we drift apart from what we perceive as the center (or as the end) there is an illusory exaggeration of the distance between it and the points close to it. The perceptual distortion of the metric structure of the line would thus be functional to a visual estimation of its center.

We can apply the same idea to the two-dimensional shapes we studied here: positions on the one-dimensional line correspond to loci inside the two-dimensional shapes. Indeed, here we observed a systematic modulation of the error comparable to the one evidenced for the line. Thus, we can generalize the idea outlined above: to establish the center of a shape we need the information supplied by its edges; once estimated, the center is maintained perceptually stable by creating a safety zone around it, where a repulsion effect is observed. In fact, the repulsion exerted by the center and by the edges of a shape warps its inner space as shown in Figs 3 and 5: observers mislocalize the inner loci nearby the center toward the edges and the loci nearby the edges toward the center, as if both the center and the edges exerted a repulsion on the neighbouring space. Such opposite repulsions reach a perfect compensation at an equilibrium point between them (Figs 2B and 4B). Furthermore, the center of a shape can be detected with minimal error, which, together with the repulsive effect it exerts on the neighbouring regions, gives the center a special perceptual status. The most obvious and ecologically plausible reason for its special perceptual status is that an accurate estimation of the center is crucial to the interaction with objects in our environment: the perceptual power of the center [28] is also confirmed by the eye-gaze tuning to the center of mass of objects [29,30,31,32,33,34,35]. Furthermore, the estimated center may act as an attentional attractor [29,31,32,34,35,36,37]. Attentional factors and eye-gaze factors normally act in synergy [38] and both account for a special status of the center.

We conducted Experiment 3 to better assess the role that overt visual attention may play in the systematic warping of the space enclosed by a shape. Eye-movements recordings are in line with the idea of the perceptual power of the center of a shape. In Experiment 3, when only the circle is presented without any target inside it, participants look at the center or at positions very close to the center of the circle. Fixations during the following phases of the trial sequence, when participants have to assess the target location and then reproduce it, are consistent with the hypothesis that the position of a point inside a shape is evaluated relative to visible geometrical landmarks, as the shape edges, or other perceptually salient (though not immediately visible) geometrical elements, as the shape center and its horizontal axis. Participants' behavior (as illustrated in Fig 7) strongly supports the interpretation that the target position does not have an absolute representation but is represented relative to specific elements of the given shape. Overall, our results suggest that identifying loci inside a shape is a complex relational process which organizes immediately visible geometrical elements as edges with represented geometrical elements as the center and the horizontal axis in a relational perceptual structure. This reminds us of Wertheimer's claim that the perceptual value of any point of a visual shape is a function of its total structure. Such a complex process does not exclude attentional components. However, it does not support their substantial role in the phenomenon we studied either: the observed perceptual distortion of the space within a figure cannot be exhaustively explained in terms of attentional deployment, as participants fixated all over the diameter of the figure both when they were first presented with the target stimulus and when they provided the response. In addition, Wardak and colleagues [27] showed that, if anything, allocation of both overt and covert attention to a point in space attracts (rather than repel, as in our experiments) position estimates towards the attentional locus in a bisection task. Isolating possible attentional factors is certainly difficult given our experimental paradigm: at variance with what typically happens in studies aiming to investigate attention, where the duration of the stimulus display is in the range of tens of milliseconds (e.g. [27,39]) and fixation is constrained to a specific point on the display, in our experiments participants were left free to visually explore the stimulus for 1.5 seconds before it disappeared. In fact, our aim was to examine how the circle and the square figures perceptually organize the space they delimit in conditions that could reasonably resemble real-life conditions, while permitting to control for possible confounding variables. Therefore, we chose not to constrain fixation and to allow sufficient time to explore the stimulus. Indeed, as Findlay and Ghilchrist [40] noted, visual attention cannot be easily dissociated from perception in ecological conditions of active vision, were the observer can voluntarily direct his/her eyes towards salient elements of the visual scene: in such conditions covert attention can play only a minor role. On the basis of our experiments, we can conclude that the warping of the visual space enclosed by a shape is not strictly attention-dependent, as observers showed rather distributed fixation patterns along the whole horizontal diameter of the figure (and beyond). Further research is needed to better assess the role of attention in the organization of space delimited by a figure.

The nice modulation of error we observed in the space enclosed within a circumference and within a square is remarkably consistent across observers, so that it may be likely governed by some perceptual organization rule, which can be described as a force vector field (see Introduction). A vector field is used in physics to describe the forces exerted by one object on another. In our case, we can consider the edges and the center of the shapes as the two interacting elements which generate, in estimating the loci within a shape, a ruled modulation of error. According to the traditional description of force vector fields in the perceptual domain [10,16], the distribution of forces within a field is governed by an economy principle of minimal energy expenditure [41]. Here we propose that the force field organization of a shape is functional to the identification of its center. In this perspective, the localization of any point within a figure is a function of the global structure of the figure, which can be described in terms of a force field. This force field is imposed by the figure structure as a consequence of the determination of its center. In other words, when we observe a figure we perceive its inner structure as warped because the identification of its center is automatically triggered. It is worthwhile noting that the proposed interpretation of the data is a purely descriptive one: our empirical data show a continuous warping of the perceptual space enclosed by a shape, which can be described in terms of vector fields independent of the origin of these vector fields. In other words, in our view attention may play a role in the determination of Gestalten, which are nevertheless Gestalten at the perceptual level (i.e. in the way they look like and organize perception).

Previous research [14,42]investigated observers’ ability to reproduce the position of a point within a field. Attneave [20] presented spots of light within a circular white screen and Stadler and colleagues [14]asked their observers to reproduce the position of a point within a DIN A4 sheet of paper. In both cases, the area under investigation was the field itself, at variance with our study, where the shape was defined by a plain outline within a homogeneous field. Our results are in line with the ones of Attneave [20] insofar as he found that points were displaced away from the edges and away from the center, with minimal error in an intermediate region between center and periphery. At variance with us, however, he further observed a repulsion away from the horizontal and vertical diameters, so that points tended to be reproduced toward the middle of the quadrant within which they were presented. This discrepancy with our results could be explained by the fact that half of his observers were presented with points on the left side of the circular screen, the other half with points on the right: this may have induced observers to use the vertical and horizontal diameters as subjective landmarks in a large and relatively unstructured field. Stadler and colleagues [14]found that observers tended to reproduce the points toward four “attractor” locations nearby the four angles and systematically away from the center. Based on this result, the authors claimed that the center of their rectangular area is the point of highest instability. Instead, our data show that the center of a shape is reproduced very accurately: this is in agreement with Arnheim’s observations in the domain of visual arts that the center of a figure represents the most stable position [43,44,45]. In our perspective, the repulsive effect of the center on the points nearby underpins its accurate identification and can be viewed as the result of a vector force field determined by the geometric structure of the shape. Indeed, we propose that the perceptual force field exerted by a Gestalt is functional to the identification of its center.

The work of Psotka [13] implicitly posed an experimental question closely related to our experiments. In his study, Psotka aimed to test Blum's theory [46,47] that visual shapes have an internal geometrical structure that he dubbed "theoretical field process". The internal geometrical structure of a shape can be intuitively described by the physical model of the "grass fire": if we take a rectangular field of dry grass and simultaneously light a fire along its perimeter, the fire that burns the grass at constant velocity extinguishes itself along the symmetry axes of the shape defined by Blum's geometry. In order to test the psychological validity of Blum's idea, Psotka employed several instances of geometrical shapes and asked a very large number of observers to draw a single point inside each shape at their favorite location. He then assembled all the points he collected within a single figure (per shape) and found that their distribution happened to match the symmetrical axes proposed by Blum as the specific geometrical descriptors of each shape. These results support the idea that every shape has its own internal structure, or, in our framework, its specific vector field.

In conclusion, our results support the idea of structural interactions between perceptually salient elements of a figure (center and outline) and can be nicely framed in the vector field approach proposed by the Gestalt psychologists for visual phenomena. Our experiments suggest that the perceived position of any point inside a visual shape is a function of the total shape characterized as a force vector field determined by the interaction between the edges and the center. The systematic misperception of the inner points of a shape induced by the vector field is likely functional to the correct identification of the center of the shape.

## Supporting Information

S1 Table Set of data collected for the present work; the Excel file contains data collected in Experiment 1, 2 and 3, both for Radial and Tangential Errors, in separate sheets. Numerical values represent localization errors made by each participant on each trial; all values are in pixels. Data are hierarchically organized by the three within-subjects factors (Radius, Location and Repetition–column values). Each row refers to each one of the six participants. In each sheet, an image presents the reference system for radii and locations numbering (clockwise for radii; form center to periphery for locations).
(XLSX)
